# Supplementary material for: Spatial index relating urban environment to health lifestyle and obesity risk in men and women from different age groups
Source: PLoS One. 2020 Mar 12;15(3):e0229961. doi: 10.1371/journal.pone.0229961 (PMC7067401; doi:10.1371/journal.pone.0229961)
Supplement: S2 Table — (DOCX) [file pone.0229961.s002.docx]

***PORTUGUESE VERSION***

**QUESTIONÁRIO HÁBITOS DE VIDA, DADOS DE SAÚDE E ATIVIDADE FÍSICA HABITUAL**

Nome/Voluntário:____________________________________________________________________

Avaliador(a): ______________________________________ Data da coleta: ______/______/_______

Local: _________________

1- Dados Antropométricos

**1.1** Data: : ______/______/___________ **1.2** Nome:_________________________________

**1.3** Local ( ) Diacuí ( ) JK | **1.4** SEXO: ( ) M ( ) F | **1.5** Idade: __________

**1.6** Peso: ________ **1.7** Altura: _________ **1.8** C.A.: __________

**1.9** IMC: ________

2- Comportamento e hábitos de vida

**3.1** Tabagismo: ( ) Fumante atual ( )Ex fumante ( ) Às vezes ( ) Nunca fumou

**3.2** Álcool: ( ) Sim ( **2** ) Não | **3.3** Consumo Semanal: ( ) 1 vez ( ) 2 vezes ( ) 3 vezes ( )Maioria dos dias

**3.4** Faz dieta: ( ) Sim ( ) Não

**3.5** Você controla o consumo de Sal? ( ) Sim ( ) Não

**3.6** Usa algum medicamento? ( ) Sim ( ) Não

4- Perfil e Pratica de Atividade Física (AF) Habitual

**4.1** Você pratica A.F. regularmente neste local? ( ) Sim ( ) Não| **4.2** nº vezes/semana:( ) 1 ( ) 2 ( ) 3 ( ) ≥ 3 ( ) Todos os Dias

**4.3** Modalidade: ( ) Caminhada ( ) Corrida ( ) Musculação ( ) Hidro/Natação ( ) Esportes ( ) Outros:

**4.4** Qual local pratica? ( ) Academia ( ) Ar livre (parque, rua) ( ) Em casa ( ) Clube ( ) Clínica ( ) Outros

**4.5** Qual seu principal objetivo com a pratica de atividade física? ( ) Saúde ( ) Estética ( ) Social ( ) redução do Estresse ( ) Lazer

**4.6** Há quanto tempo frequenta este local? ( ) Primeira vez ( ) Poucas semanas ( ) Um mês ( ) 1-3 meses ( ) 3-6 meses ( ) 6-9 meses ( ) 9-12 meses ( ) ≥ 1 ano

**4.7** Qual a duração (tempo) de A.F que realiza? ( ) +1hora ( ) - 1 hora ( ) 2-3 horas

**4.8** Você aquece antes da A.F.? ( ) Sim ( ) Não **4.9** Você alonga antes da A.F.? ( ) Sim ( ) Não **4.10** Você alonga após a A.F.? ( ) Sim ( ) Não

**4.11** Você tem orientação de profissional de Educação Física para praticar A.F no neste local? ( )Sim ( ) Não

**4.12** Considera Importante orientação de um especialista? ( ) Sim ( ) Não

**4.13** Você utiliza os aparelhos da Academia ao ar livre? ( ) Sim ( ) Não

***ENGLISH VERSION***

**QUESTIONNARIE OF HABITS OF LIFE, HEALTH AND PHYSICAL ACTIVITY**

Name: ____________________________________________________________________________

Researcher: ____________________________ Attended at: ______(dd)/______(mm)/_______(yyyy)

Local: _________________

1- Anthropometric report:

**1.1** Date: ______/______/___________ **1.2** Name:_________________________________

**1.3** Local ( ) Diacuí ( ) JK | **1.4** SEX: ( ) M ( ) F | **1.5** Age: __________

**1.6** Weight: ________ **1.7** Height: _________ **1.8** Ab.C: __________

**1.9** BMI: ________

2- Habits of life

**3.1** Smoking: ( ) Frequent ( ) Not Frequent ( ) No longer smoking ( ) Never

**3.2** Alcohol: ( ) Yes ( **2** ) No | **3.3** Weekly intake: ( ) once ( ) twice ( ) three times ( ) more than three

**3.4** Diet: ( ) Yes ( ) No

**3.5** Control of salt intake? ( ) Yes ( ) No

**3.6** Drug therapy: ( ) Yes ( ) No

4- Physical Activity (PA) Practice

**4.1** Do you practice PA at this place? ( ) Yes ( ) No| **4.2** Sections per week: ( ) 1 ( ) 2 ( ) 3 ( ) ≥ 3 ( ) daily

**4.3** Modality: ( ) Walking ( ) Running ( ) Weight Training ( ) Water Exercise/Swimming ( ) Sports ( ) Others:

**4.4** Place of practice: ( ) Gyn ( ) Open urban spaces (park, street) ( ) At home ( ) Athletic associations ( ) Clinical rooms ( ) Others:

**4.5** What is your main objective with the practice of physical activity? ( ) Health ( ) Aesthetics ( ) Socialization ( ) Relieve the stress ( ) Leisure

**4.6** How long have you been exercising at this place? ( ) First time ( ) Some weeks ( ) One month ( )1-3 months ( ) 3-6 months ( ) 6-9 months ( ) 9-12 months ( ) ≥ 1 year

**4.7** Physical activity duration per section: ( ) +1 hour ( ) - 1 hour ( ) 2-3 hours

**4.8** Do you warm up before P.A.? ( ) Yes ( ) No **4.9** Do you stretching before P.A.? ( ) Yes ( ) No **4.10** Do you stretching after P.A.? ( ) Yes ( ) No

**4.11** Have you supervision from Physical Education professional for the practice of P.A. at this place? ( )Yes ( ) No

**4.12** Do you consider professional supervision important? ( ) Yes ( ) No

**4.13** Do you perform exercise in public gymnastic equipment? ( ) Yes ( ) No
