# Supplementary material for: Spatial index relating urban environment to health lifestyle and obesity risk in men and women from different age groups
Source: PLoS One. 2020 Mar 12;15(3):e0229961. doi: 10.1371/journal.pone.0229961 (PMC7067401; doi:10.1371/journal.pone.0229961)
Supplement: Attachment — Submitted filename: Response_to_Reviewers_PONE_D-19-29357.docx [file pone.0229961.s007.docx]

Letter to Reviewers of PONE-D-19-29357 (Spatial index relating urban environment to health lifestyle and obesity risk in men and women from different age groups).

Dear Robert Siegel (Academic Editor of PLOS ONE)

The authors appreciate the time of the reviewers in revising the manuscript carefully. The authors also thank the reviewers for the interesting comments, which will improve the quality of the manuscript. All revised text is shown in red.

**Editor:**

**#1** *- If you would like to make changes to your financial disclosure, please include your updated statement in your cover letter.*

Response: No, we didn’t make any changes.

**#2** - *To enhance the reproducibility of your results, we recommend that if applicable you deposit your laboratory protocols in protocols.io, where a protocol can be assigned its own identifier (DOI) such that it can be cited independently in the future. For instructions see: http://journals.plos.org/plosone/s/submission-guidelines#loc-laboratory-protocols*

Response: Thank you to remember us about the possibility to include the protocol on “protocols.io”. We included it, and that is the register number: dx.doi.org/10.17504/protocols.io.bb3jiqkn.

**Journal Requirements:**

**#1**. *Please ensure that your manuscript meets PLOS ONE's style requirements, including those for file naming. The PLOS ONE style templates can be found at http://www.journals.plos.org/plosone/s/file?id=wjVg/PLOSOne_formatting_sample_main_body.pdf and http://www.journals.plos.org/plosone/s/file?id=ba62/PLOSOne_formatting_sample_title_authors_affiliations.pdf*.

Response: The manuscript and title page revised according to the template formats, which were downloaded from the links delivered by the editor.

**#2**. *Please include additional information regarding the survey or questionnaire used in the study and ensure that you have provided sufficient details that others could replicate the analyses. For instance, if you developed a questionnaire as part of this study and it is not under a copyright more restrictive than CC-BY, please include a copy, in both the original language and English, as Supporting Information. In addition, please provide any details of pre-testing of this questionnaire - i.e. how many participants were involved and from where were they recruited.*

Response: The Portuguese and English versions of the questionnaire designed for the present study are available in “Supporting Information” and labeled as S2-Table. Additionally, the authors would like to declare the authorship of such questionnaire, the full-version of which is present in the S2-Table. This questionnaire has been applied as a survey procedure to inquire about life habits among persons practicing physical activity in open urban spaces, on behalf of the social program of the Federal University to support citizens from Jataí to reduce obesity and cardiovascular risks. This program started in 2016 assisting approximately 300 per year, from which were derived academic studies in both undergraduate and graduate levels.

**#3**. *Please be wary of causal statements in your manuscript, including "the adoption of healthy life habits (practicing regular PA and avoiding smoking and excessive alcohol consumption) have a protective effect". Such statements cannot be supporting following a study of this design.*

Response: The author has altered the sentence, adjusting the statement according to the information acquired and modeled: " … suggesting that the reported healthy life habits (practicing regular PA and avoiding smoking and excessive alcohol consumption), with the SUHI weighting to correlate negatively with BMI, is a reasonable inference to an integrative effect between urban space, life habits and physical activity able to reduce sedentary and obesogenic tendencies, …”

**#4.** *We note that Figure 1 in your submission contains satellite images which may be copyrighted. All PLOS content is published under the Creative Commons Attribution License (CC BY 4.0), which means that the manuscript, images, and Supporting Information files will be freely available online, and any third party is permitted to access, download, copy, distribute, and use these materials in any way, even commercially, with proper attribution. For these reasons, we cannot publish previously copyrighted maps or satellite images created using proprietary data, such as Google software (Google Maps, Street View, and Earth). For more information, see our copyright guidelines: http://journals.plos.org/plosone/s/licenses-and-copyright.*

Response: Figure 1 is not an image under license rights, neither does it need permission to be published or have unrestricted use. Such an image is a map that is an output from the present study, as well as other data produced in tables and text. The cartographic database is numerical in nature, which was obtained from open-access repositories supplied to governmental agencies, as properly referred in the references [17-18]. Furthermore, numerical information was integrated into geographical information system (GIS) software to produce information in map format. The authors have detailed this procedure further in protocols.io. In the Figure 1 caption, the authors include the following text: “Note: cartographical output produced by the authors, with original content modelled from open-access information not requiring license, permission and copyright declaration”.

**#5.** *Please ensure that you refer to Figure 1 in your text as, if accepted, production will need this reference to link the reader to the figure.*

Response: Fig. 1 has been properly referred on lines 335 and 339. The author would like to thank the editor for this observation.

**#6.** *Overall a very interesting study. An overall and simple explanation of the statistical approach would be helpful to many readers.*

Response: The authors have included a brief explanation concerning the statistical procedure and what is expected in the results from each analysis. All new information has been included in the Statistical Treatment section. The authors hope that such insertions will make the technical language of the statistical procedures more common.

**Review Comments to the Author**

*Reviewer #1:*

**#1.** *This is an interesting paper as it seeks to develop a quantification tool to assess environmental factors that may contribute to activity levels and health lifestyle. This was a concept explored by the Australian group Leslie E et al., Health and Place 2007 but has never really been developed. The tool used four main indicators i.e. population density, road connectivity, area of green spaces and land incline in the calculation of the spatial index.*

Response: The authors appreciate this comment and the reviewer time dedicated to accomplish the revision. Yes, this study coupled expertise from two different fields (Spatial and Health Sciences) into an attempt to clarify how urban features are integrated to healthy life habits.

**#2.** *Relevant ethical approval appears to have been obtained for the study*.

Response: Yes, it has. This study launched the sampling process after ethical approval, which was referred on line 158. This is an obligatory phase for studies involving humans in Brazil. Regularly, studies of such nature should be submitted to the National Counsel of Ethics in Research through an on-line platform (<http://plataformabrasil.saude.gov.br/login.jsf>), which are thereafter redirected to local committees of the University where the research must be approved before it is developed.

**#3.** *It is not clear how recruitment to the study was decided however to some degree it appears a biased population was obtained, as the study only sampled those who were outdoors using park or outdoor spaces. The study results may have been more powerful if this was compared with a control group of people approached within office spaces or homes within those areas. Alternatively comparing this population against a similar cohort from Goiania to verify results.*

Response: The authors would like to thank the reviewer for this comment. The strategy applied to sampling the information consisted of approaching potential participants while they are arriving or leaving open public spaces, which have urban or natural conditions to support physical activity and other types of activities not related to exercise. Although many of these potential participants may be an occasional practitioners, or just visitors spending their free time walking around the site, therefore enabling us to consider them as the control population, we are in agreement with the reviewer as the data collection tool only considered as an actual participant someone who was practicing some type of physical activity at the site. Thus, the authors considered to insert a comment advising about such limitation of the actual approach and suggest the inclusion of non-physical activity practitioners (or population that have never used parks or other urban public spaces) in the sample of future studies. Please see lines p. 444 – 451.

**#4.** *The paper is well written and statistical methods appear robust.*

Response: The authors would like to thank the reviewer for recognizing the proficiency of English language usage and the robustness of analytical procedures applied to assure the confidence of the results.

**#5.** *Further description is required regarding how the actual axiological/anthropometric measurements were taken (e.g. number of observers at any one time) and how standardization was preserved to prevent significant inter or intra observer error. Some increased clarity on this needs to be included.*

Response: The author thanks the reviewer for the information about the need for a detailed description of anthropometric measurement. Please see lines 196 to 205 for the complete information.

**#6.** *Increasing BMI in the male group seems to correlate better with abdominal circumference than in the female group. This may have confounded the significance in result obtained in the two gender groups. A better measure may have been to not just look at BMI but using a portable bioimpedance scale to measure percentage fat versus muscle mass.*

Response: The authors thank the reviewer for this comment. We are in agreement with the reviewer, since the improvement of the robustness of obesity assessment (by using bio-impedance or dual X-ray absorptiometry) certainly will ensure evidence for the role or spatial urban spaces to stimulate healthy life habits and reduced risks of obesity, regardless of gender and age group. Thus, the authors considered to insert a comment advising about such limitation of BMI and suggest the inclusion of tools to assess body composition in the sample, of future studies. Please see lines p. 458 – 461.

**#7.** *Although brief assessment of health risks was made in the study population such as smoking, dietary control and alcohol consumption, it is a shame that brief assessment of actual metabolic comorbidities such as heart disease and diabetes was not done and included in the analyses.*

Response: The authors would like to thank the reviewer for this comment. We are in agreement with the reviewer, since a clinical test for the diagnosis of diabetes and associated comorbidities will provide an assertive status of the participant’s health. Thus, the authors considered to suggest the inclusion of such analysis in future studies. Please see lines p. 458 – 461.

**#8.** *The discussion did effectively identify some of the limitations of the study, particularly to address the possible effect that the limited level of higher-intensity cardiovascular activity (running) was in the group and may have been contributory to the lack of statistical significance in the results.*

Response: The authors agree with the reviewer about the low conditioning level of the cardiorespiratory system would probably be a constraint on the level of correlations, mainly among female participants. The main determinant would be the accommodation of the training overload, since a physical activity schedule is usually not under professional supervision. Only cardiorespiratory assessment (e.g. maximal oxygen uptake, V̇O_2max_) would evidence whether or not the conditioning level is compromised between our sampled population and limiting correlations to the risk of obesity for females and young participants, as we commented on lines 440 to 443.

**#9.** *When looking at this study along with other systematic reviews on effects of urban environments on health outcomes, it is clear that more confounders needs to be included in the measures such as actual equipment available for activity, indoor exercise spaces particularly in urban areas and population demographic that may be affected by ethnicity and other genetic factors.*

Response: The authors agree with the reviewer that it is highly advisable for future studies to analyze the effect of spatial urban features in a population other than that included in the present study. Such recommendations are described on lines 449-453. The authors realize that a population engaged in physical activity at other places (not necessary public), or those not engaged on physical activity (users of public urban spaces, or not) should be included in future studies to verify whether spatial urban features also contribute to stimulate life habits among them, therefore ratifying (or not) the importance of urban space for the health of population.

**#10.** *Nevertheless, this paper is original in its approach and will almost certainly act as a study that will generate further research in the area.*

Response: The authors also hope that this research will provide the basis for future studies aiming at clarifying how spatial urban features affect human health and well-being. Thank you so much for all recommendations and advice.

*Reviewer #2:*

**#1.** *Very interesting paper. Having an urban environment that promotes exercise is important and encourages exercise. However, I was not surprised that there was no effect on BMI given the little impact that exercise plays in weight loss and changes in BMI. Especially when the main mode of activity is walking and given how little calories it burns when compared to other more intense exercise. Many studies show eating habits and genetic predisposition play a much larger role in weight loss and changes in BMI.*

Response: The authors appreciate this comment and the reviewer time dedicated to accomplish the revision. Also, the authors would like to comment that the new study from our research group included dual X-ray absorptiometry for body composition assessment and a complete nutritional report to be a complementary analysis regarding the role of urban spatial features on health life habits, and consequently on the reduction of the risks of obesity and cardiovascular diseases. Therefore, having this reviewer comment as the base, the authors are sure that the new approach is a relevant research matter.

**#2.** *It would have been nice to look at body composition as well to see if the higher BMI's were because of excess body fat or higher muscle mass in the more active population.*

Response: The authors would like to thank the reviewer for this comment. We are in agreement with the reviewer, since the assessment of body composition by using bio-impedance or dual X-ray absorptiometry, certainly will discriminate BMI major determinants (fat mass or fat free mass) and contribute to the discussion about the risk of obesity parametrized to BMI values, and the lack of correlation for female participants and young males. This has been pointed out as suggestions for futures studies on lines 458 to 461.

**#3.** *I found the statistical analysis very confusing but the paper was overall pretty easy to read and understand*.

Response: The authors have included a brief explanation concerning the statistical procedure and what is expected in the results from each analysis. Please see lines 253 to 295. The authors hope that such insertions will make the technical language of the statistical procedures more common.
